# Supplementary material for: Multistage Detection of Tetrodotoxin Traces in Diodon hystrix Collected in El Salvador
Source: Toxins (Basel). 2023 Jun 25;15(7):409. doi: 10.3390/toxins15070409 (PMC10467132; doi:10.3390/toxins15070409)
Supplement: Supplementary file 1 [file toxins-15-00409-s001.zip › toxins-2384667-supplementary.pdf]

Supplementary Materials

Multistage detection of Tetrodotoxin traces in *Diodon hystrix* collected in El Salvador

Juan Carlos Fuentes-Monteverde [1,2,†], Marvin J. Núñez [3,†], Oscar Amaya-Monterosa [4], Morena L. Martínez [3], Jaime Rodríguez [1,*] and Carlos Jiménez [1,*]

[1] CICA—Centro Interdisciplinar de Química e Bioloxía and Departamento de Química, Facultade de Ciencias, Universidade da Coruña, 15071 A Coruña, Spain; jufu@mpinat.mpg.de

[2] NMR Based Structural Biology, MPI for Multidisciplinary Sciences, Am Fassberg 11, 37077 Göttingen, Germany

[3] Laboratorio de Investigación en Productos Naturales, Facultad de Química y Farmacia, Universidad de El Salvador, San Salvador 01101, El Salvador; marvin.nunez@ues.edu.sv (M.J.N.); morena.martinez@ues.edu.sv (M.L.M.)

[4] Laboratorio de Toxinas Marinas, Escuela de Física, Facultad de Ciencias Naturales y Matemática, Universidad de El Salvador, San Salvador 01101, El Salvador; oscar.amaya@ues.edu.sv

* Correspondence: jaime.rodriguez@udc.es (J.R.); carlos.jimenez@udc.es (C.J.); Tel.: +34-881012173 (J.R.); +34-881012170 (C.J.)

† These authors contributed equally to this work.

Contents

Samples of Diodon hystrix 4

Figure S1. Female specimen of *Diodon hystrix*. 4

Figure S2. Dissected specimens of *Diodon hystrix*. 4

Sample purification 4

Figure S3. Aqueous layer ultra-filtration (Amilcon® system) of the sample (Left) and sample TTX concentration via manual pass-through a Sep-Pak Plus (C18 Cartridges) Waters (Right). 4

Detection and quantification limit of HPLC-MS/MS-SRM 5

Table S1. Calibration curve data 5

Figure S4. TTX calibration curve for TTX of the qualitative SRM transition (320 → 302, Curve I), and the quantitative SRM transition (320 → 162, Curve II), over the calibration range used in samples of *D. hystrix* study. Linear regression curves are shown in dashed lines, slope (b) and interceptor (a) are displayed with its respective standard deviations, limit of detection (LOD: 0.076 μg mL^{-1}) and limit of quantification (LOD: 0.32 μg mL^{-1}) were computed analytically from Curve I and II, indicated in the figure and the inset respectively, the statistic $S_{y/x}$ (S_B), and the estimated standard deviation of the blank (S_B-I = 33139.83; S_B-II = 31105.52). 6

HPLC-MS/MS-*SRM* analysis 7

Figure S5. HPLC-MS/MS-SRM Chromatograms of TTX standard (**a**), and muscle undefined sample (**b**). Arrows show the expected transition. The expected transition ion ratio (TIR) between the two quantifications ions was not found in the sample, presumably due low sample concentration. 7

HPLC-HRFTMS analysis 7

Figure S6. HPLC-ESI(+)-HRMS (SIM mode) of tetrodotoxin standard. Total ion current (**a**), chromatogram extract mass range *m/z* 320.00-320.20 (**b**) and high-resolution mass spectra (**c**) 7

Figure S7. Total ion current of the HPLC-ESI(+)-HRFTMS (SIM mode) of FS sample of *D. hystrix* 8

Figure S8. Isotope Abundance analysis of HPLC-ESI(+)-HRFTMS (*SIM* mode) study of FS sample (*D. hystrix*). Isotope abundace list generated by Thermo Xcalibur V 3.0 at different retention times (**a**). Calculation of experimental Relative Isotope Abundance (RIA_{exp}) (**b**). Theoretical isotope abundace list of TTX automatically generated by ChemDraw V 20.1 (**c**), and calcualtion of Relative Isotope Abundance (RIA_{error}) 8

Figure S9. Overlapping of the HPLC-ESI(+)-HRFTMS extracted ion chromatogram of TTX standard (Black peak) and Female Skin (FS) sample (Red peak) under full scan. 8

Figure S10. HPLC-ESI(+)-HRFTMS (*SIM* mode) analysis of male liver sample of *D. hystrix*. High-resolution mass spectra at diferent retentions times (**a**-**c**) 9

HPLC-HRFTMS² analysis 9

Figure S11. HPLC-ESI(+)-HRMS² of tetrodotoxin standard, obtained on a linear-trap quadruple-Orbitrap XL MS. Extracted ion chromatogram from *m/z* 320.00 → 302.50-301.5 (**a – above**). Extracted ion chromatogram from *m/z* 320.00 → 162.50-161.50 (**a – below**). Fragmentation of the ion eluted at t_R 28.9±0.1 min, shows the characteristic fragments at *m/z* 302.09799 (Δ *m/z* = 0.96 ppm); which indicates the losing of a molecule of water on tetrodotoxin molecule (**b**). Second fragmentation of the ion shows the characteristic fragments at *m/z* 162.06595 (Δ *m/z* = 1.48 ppm); which suggests the presents of the distinguishing group (5-8)-hydroxyquinazolin-2-amonium, commonly found in TTX fragmentation (**c**). 9

Parameters of LC/MS Analysis in MZMINE V2.21 10

Table S2. Range of element used during Tetrodotoxin dereplication 10

Samples of Diodon hystrix

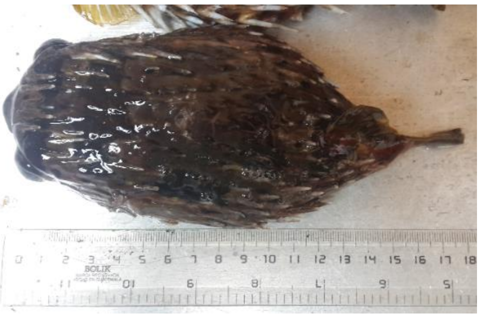

Figure S1. Female specimen of *Diodon hystrix.*

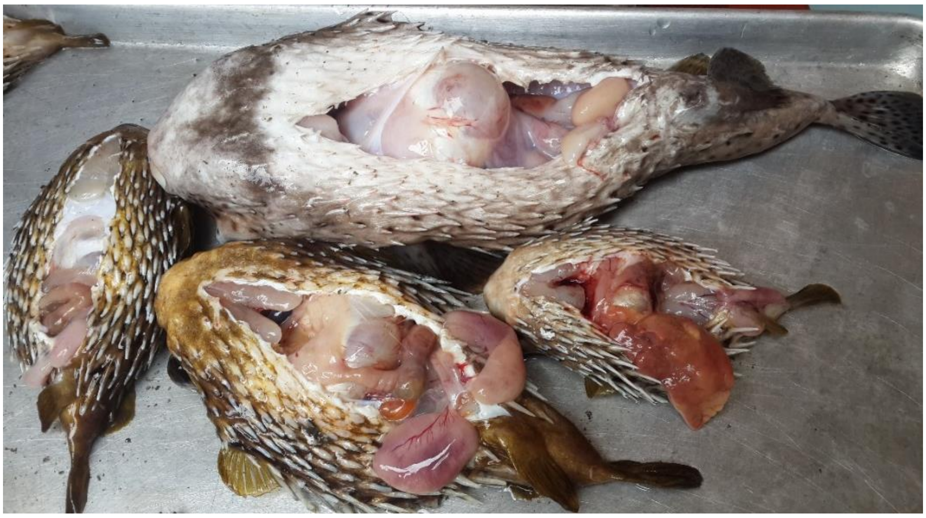

Figure S2. Dissected specimens of *Diodon hystrix*.

Sample purification

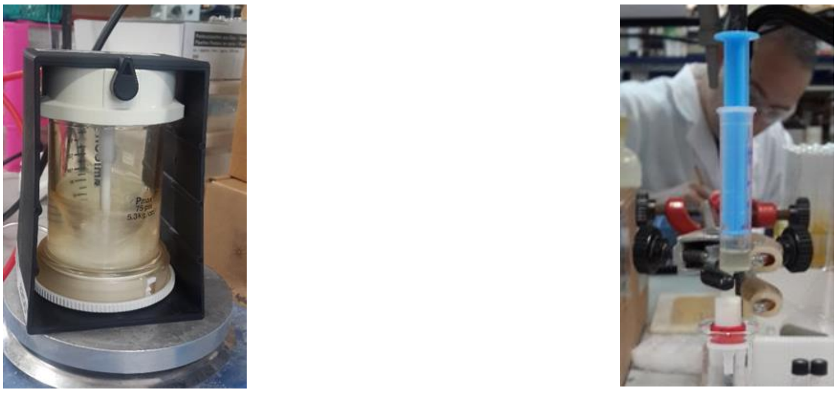

Figure S3. Aqueous layer ultra-filtration (Amilcon® system) of the sample (Left) and sample TTX concentration via manual pass-through a Sep-Pak Plus (C18 Cartridges) Waters (Right).

Detection and quantification limit of HPLC-MS/MS-SRM

The *limit of detection* (LOD) and *quantification* (LOQ) were calculated as follows: Detection and quantification curves were built by plotting the area of both 320 → 302 and 320 → 162 transitions, over the calibration range used in samples of *D. hystrix* study; named as Curve I and Curve II respectively (See **Figure S4**). Both curves were adjusted by linear regression to the general form $y = bx + a$, where b is the *slope* and a is the *interceptor*. The sensitivity of the method is defined as the b. LOD was computed with the parameters derived from Curve I, while LOQ was estimated from values obtained from Curve II; using the **equations 1** and **2,** respectively. Where y_b, is the *blank signal response*, precisely estimated from the regression curve as the *interceptor* (a-I = 36136.57 and a-II = 27978.10), and S_B is the *standard deviation of the blank*, which estimates the random error in the y-direction. S_B is equal to the statistic $S_{y/x}$ and computed by **equation 3** (S_B-I = 33139.83 and S_B-II = 31105.52). The *standard deviation of the slope* (S_b) and *standard deviation of the intercept* (S_a) were computed using **equations 4** and **5**, respectably. [1].

Equation legend, in order of aperiance: LOD, is the limit of detection; LOQ, is the limit of quatification, y_b, is the blank signal response; $S_{y/x}$ (S_B), is the standard deviation of the blank; y_i, each of the m/z areas corresponding to the TTX serial dilution; $\hat{y}_i$ computed m/z areas using the fitted curve ($y = bx + a$); x_i, each of the concentration that covers the full range of the calibration curve; $\bar{x}$, average of the concentration of the 9 standard samples used in the calibration curve building. S_b, is standard deviation of the slope, and S_a, is standard deviation of the interceptor

$$LOD = y_{b-I} + 3S_{B-I} \qquad \text{Eq. 1}$$

$$LOQ = y_{b-II} + 10S_{B-II} \qquad \text{Eq. 2}$$

$$S_{y/x} = \sqrt{\frac{\sum_i (y_i - \hat{y}_i)^2}{n-2}} \qquad \text{Eq. 3}$$

$$S_b = \frac{S_{y/x}}{\sqrt{\sum_i (x_i - \bar{x})^2}} \qquad \text{Eq. 4}$$

$$S_a = S_{y/x}\sqrt{\frac{\sum_i {x_i}^2}{n\sum_i (x_i - \bar{x})^2}} \qquad \text{Eq. 5}$$

Table S1. Calibration curve data

Concentracion (µg/mL)	Area m/z 1	Area m/z 2	TIR
0.0063	10605	7490	1.41_6
0.013	22757	16128	1.41_1
0.025	52920	39250	1.34_8
0.05	99870	71799	1.39_1
0.1	197500	145000	1.36_2
0.2	357800	268900	1.33_1
0.6	832800	661700	1.25_9
0.8	1060000	801100	1.32_3
1.01	1341000	991900	1.35_2
		**Avg.**	1.34
		**STD.**	0.04
		**RSE.**	3.09

TIR: transition ion ratio. Avg.: Average; STD.: Standard deviation; RSE.: Relative standard error. Identification criteria was stablished as ± 15% of the Avg. Quantification limit: 6.2 ng/mL, and minimun signal to noise ratio (s/n) = 10.

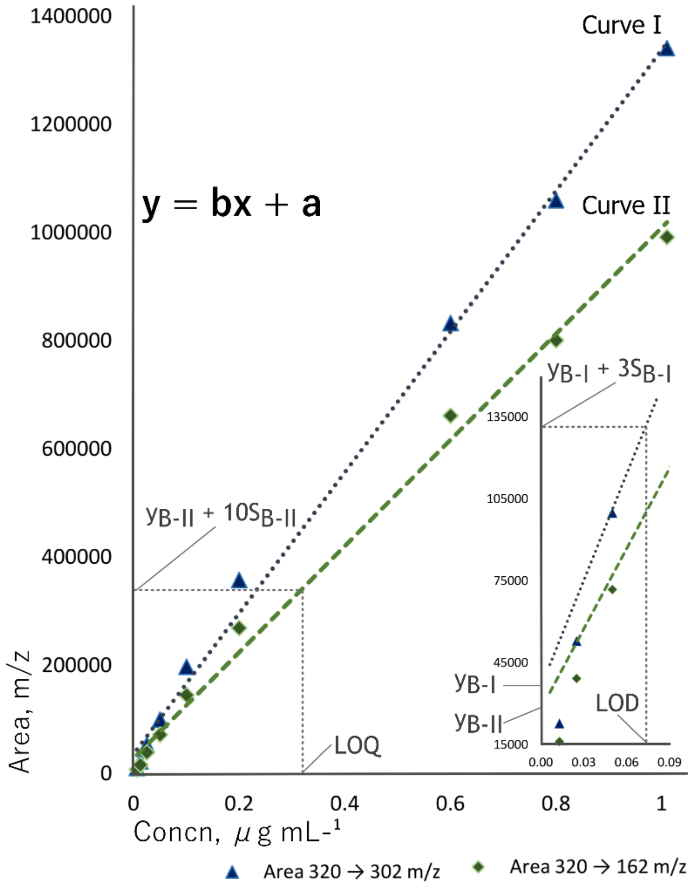

	Curve I	Curve II
$b \pm \sigma_b$	1301580.75 ± 30256.86	981158.97 ± 28399.52
$a \pm \sigma_a$	36136.57 ± 14522.71	27978.10 ± 13631.22
R^2	0.9962	0.9942
$S_{x/y}$	33139.83	31105.52
	LOD = 0.076 μg mL⁻¹	LOQ = 0.32 μg mL⁻¹

Figure S4. TTX calibration curve for TTX of the qualitative SRM transition (320 → 302, Curve I), and the quantitative SRM transition (320 → 162, Curve II), over the calibration range used in samples of *D. hystrix* study. Linear regression curves are shown in dashed lines, slope (b) and interceptor (a) are displayed with its respective standard deviations, limit of detection (LOD: 0.076 μg mL^{-1}) and limit of quantification (LOD: 0.32 μg mL^{-1}) were computed analytically from Curve I and II, indicated in the figure and the inset respectively, the statistic $S_{y/x}$ (S_B), and the estimated standard deviation of the blank (S_B-I = 33139.83; S_B-II = 31105.52).

HPLC-MS/MS-*SRM* analysis

HPLC-MS/MS analysis also shows in undefined gender muscle sample (MU) (Δt_R = 0.13, TIR = 0.21) a transitions that subsjest the presence of the toxin. TIR: transition ion ratio.

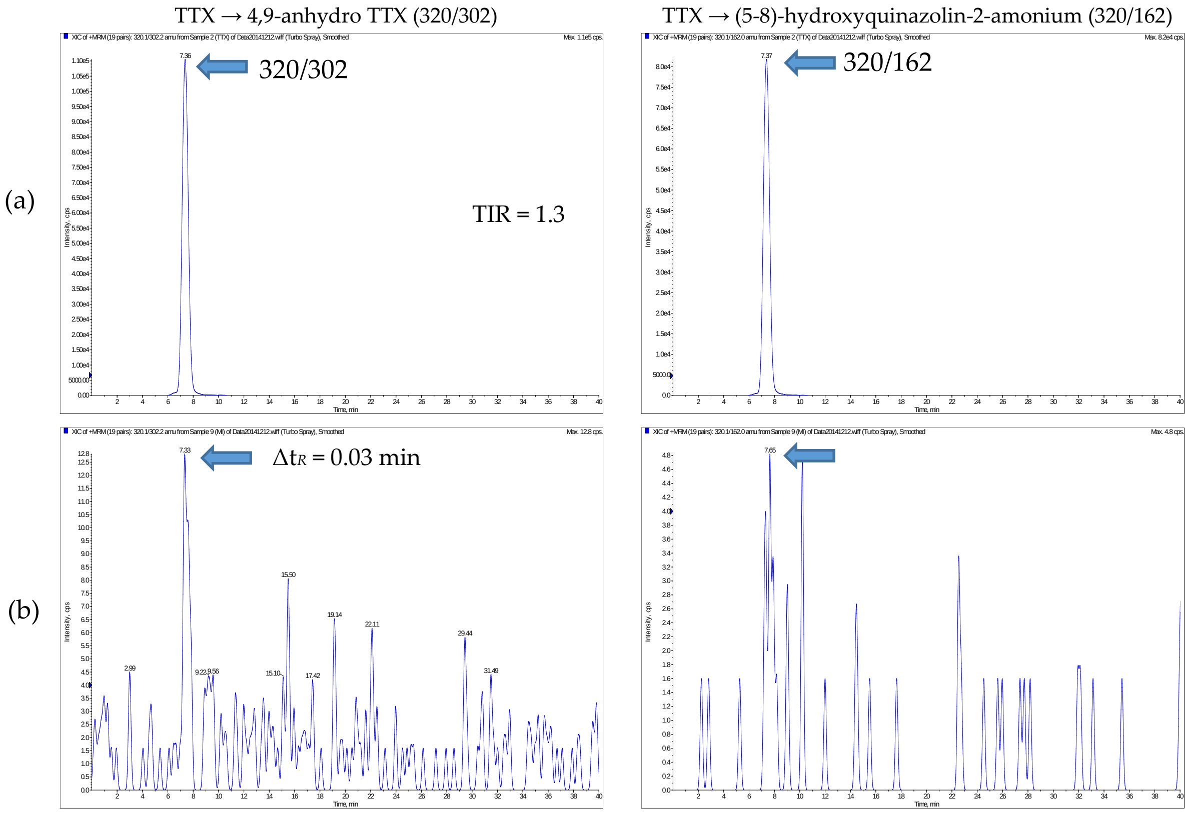

Figure S5. HPLC-MS/MS-SRM Chromatograms of TTX standard (**a**), and muscle undefined sample (**b**). Arrows show the expected transition. The expected transition ion ratio (TIR) between the two quantifications ions was not found in the sample, presumably due low sample concentration.

HPLC-HRFTMS analysis

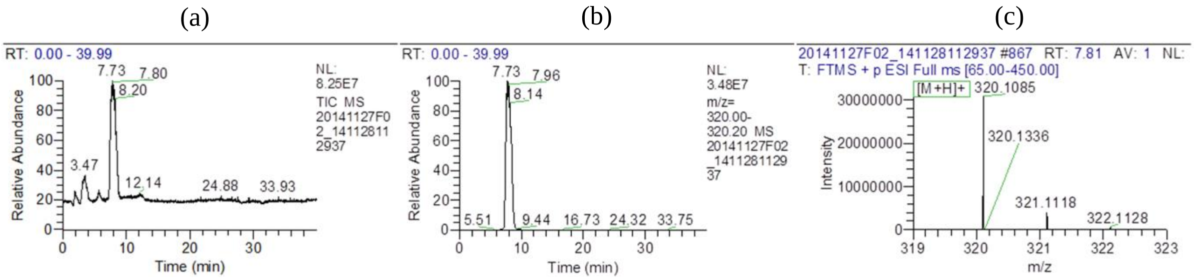

Figure S6. HPLC-ESI(+)-HRMS (SIM mode) of tetrodotoxin standard. Total ion current (**a**), chromatogram extract mass range *m/z* 320.00-320.20 (**b**) and high-resolution mass spectra (**c**)

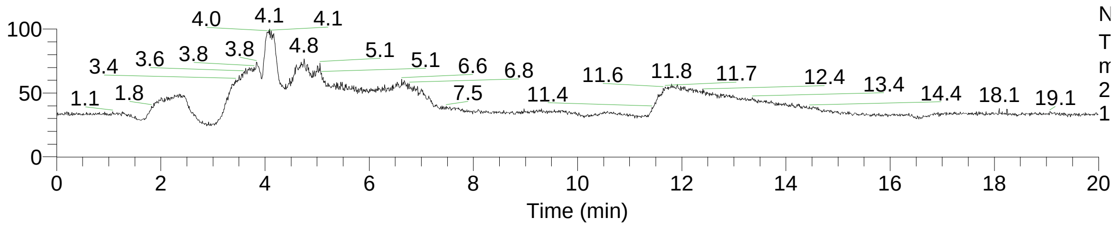

Figure S7. Total ion current of the HPLC-ESI(+)-HRFTMS (SIM mode) of FS sample of *D. hystrix*.

In that figure bellow the differences between accurate calculated mass and experimentally detected mass in ppm (Δ *m/z*) and the isotopic ion abundance ratio error (RIAerror) of M+1/M ($^{13}C_1/^{12}C$) are shown

(a)

20141127F03_141128121036#905 RT: 7.97
T: FTMS + p ESI Full ms [65.00-450.00]
m/z= 319.90-321.60

m/z	Intensity	Relative
320.10876	61723.5	100.00
320.25623	18838.9	30.52
320.49335	4523.4	7.33
321.11130	8157.9	13.22

20141127F03_141128121036#915 RT: 8.06
T: FTMS + p ESI Full ms [65.00-450.00]
m/z= 319.90000-321.60000

m/z	Intensity	Relative
320.10876	97858.3	100.00
320.25583	20896.0	21.35
321.11249	12779.2	13.06

20141127F03_141128121036#961 RT: 8.47
T: FTMS + p ESI Full ms [65.00-450.00]
m/z= 319.90-321.60

m/z	Intensity	Relative
320.10840	35510.2	100.00
320.25598	23129.6	65.14
321.11234	4661.9	13.13

(b)

$$RIA_{exp} = \frac{13.22 + 13.06 + 13.13}{3} = 13.1_4$$

(c)

Chemical Formula: C11H18N3O8+
Exact Mass: 320,10884

$^{12}C_n$ ········▸ m/z: 320.10884 (100.0%), 321.11219 (11.9%), ◂········ $^{13}C_1\,^{12}C_{n-1}$
322.11309 (1.6%), 321.10587 (1.1%)

(d)

$$RIA_{error\ (\%)} = \left|\frac{13.1_4 - 11.9}{11.9}\right| x100 = 10.4$$

Figure S8. Isotope Abundance analysis of HPLC-ESI(+)-HRFTMS (*SIM* mode) study of FS sample (*D. hystrix*). Isotope abundace list generated by Thermo Xcalibur V 3.0 at different retention times (**a**). Calculation of experimental Relative Isotope Abundance (RIA_{exp}) (**b**). Theoretical isotope abundace list of TTX automatically generated by ChemDraw V 20.1 (**c**), and calcualtion of Relative Isotope Abundance (RIA_{error})

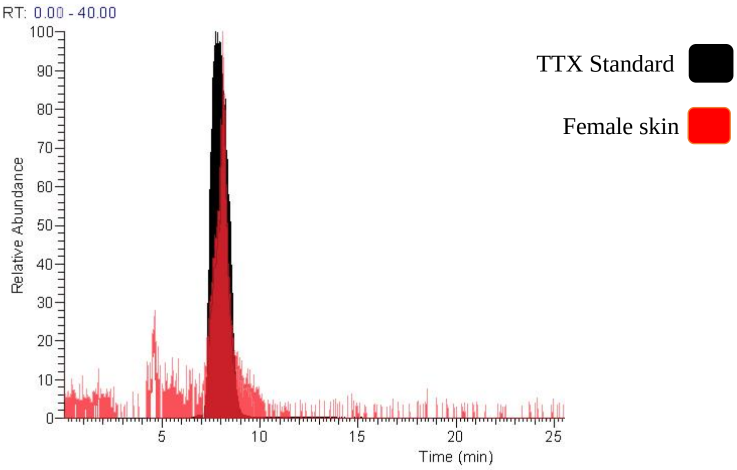

Figure S9. Overlapping of the HPLC-ESI(+)-HRFTMS extracted ion chromatogram of TTX standard (Black peak) and Female Skin (FS) sample (Red peak) under full scan.

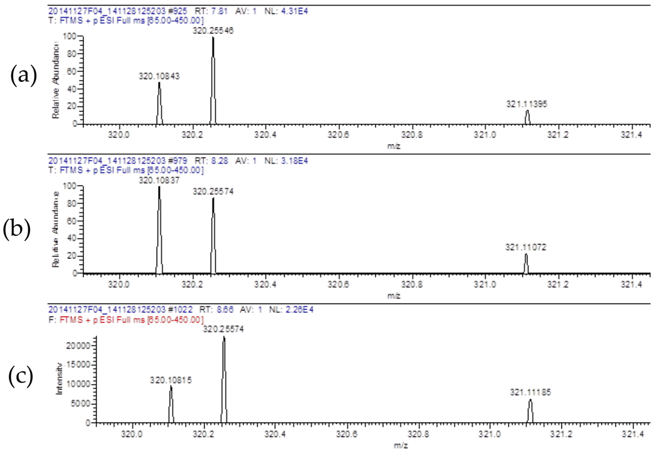

Figure S10. HPLC-ESI(+)-HRFTMS (*SIM* mode) analysis of male liver sample of *D. hystrix*. High-resolution mass spectra at diferent retentions times (**a**-**c**)

HPLC-HRFTMS^2 analysis

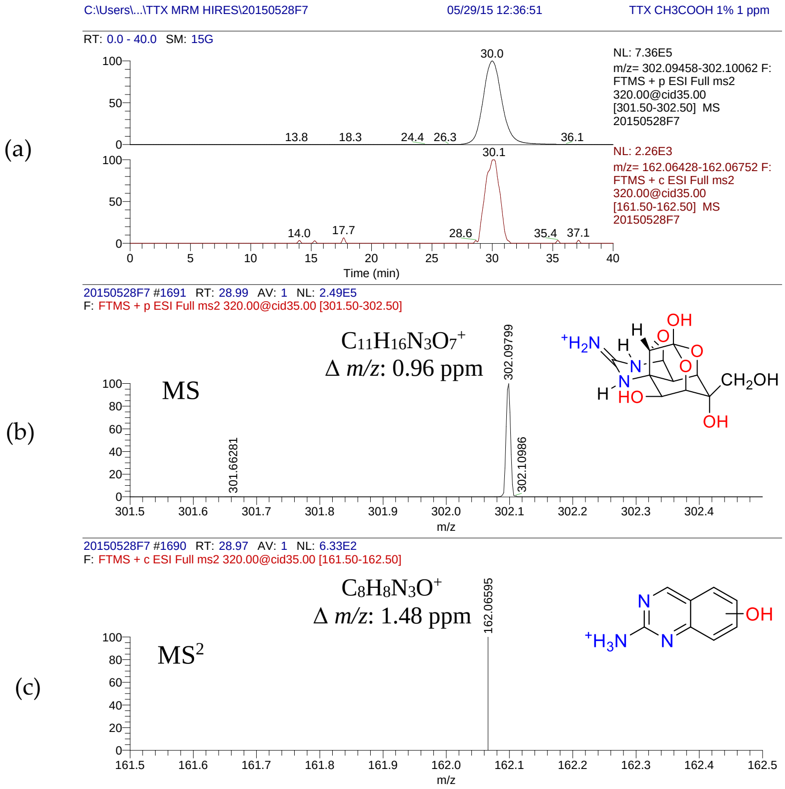

Figure S11. HPLC-ESI(+)-HRMS^2 of tetrodotoxin standard, obtained on a linear-trap quadruple-Orbitrap XL MS. Extracted ion chromatogram from *m/z* 320.00 → 302.50-301.5 (**a – above**). Extracted ion chromatogram from *m/z* 320.00 → 162.50-161.50 (**a – below**). Fragmentation of the ion eluted at t_R 28.9±0.1 min, shows the characteristic fragments at *m/z* 302.09799 (Δ *m/z* = 0.96 ppm); which indicates the losing of a molecule of water on tetrodotoxin molecule (**b**). Second fragmentation of the ion shows the characteristic fragments at *m/z* 162.06595 (Δ *m/z* = 1.48 ppm); which suggests the presents of the distinguishing group (5-8)-hydroxyquinazolin-2-amonium, commonly found in TTX fragmentation (**c**).

Parameters of LC/MS Analysis in MZMINE V2.21

Batch queue options Full Scan Mode and Tandem Ms/Ms (Options not indicated are understands to be blank. Mass tolerance used was fixed using two times the different between ttx's mass detected on the standard and the calculated value). **Mass detection**: *Scans***:** Retention time: Auto. MS Level: 1. Polarity Any. Spectrum type: Profile. *Mass detector*: Exact Mass. Noise level: 7E3. Note: Male Liver chromatogram were very noise, and was necessary a more refine approach, Mass detector (*Male liver sample*): Mass detector: Wavelet transform. **TMS shoulder peaks (Only Full scan):** Mass resolution: 10000. Peak model function: Gaussian **Ms/MS peak (Only Ms/Ms tandem):** m/z range: 162-302.5. **Baseline correction**: Chromatogram type: TIC. MS level: 1. Use m/s bins: Checked. *m/z* bin width 1**.** *Correction Method*: Asymmetric baseline corrector. Smoothing: 1E4. Asymmetry: 0.001. RT & m/z range: Auto. **Chromatogram builder**: *Scans:* Retention time: 6.0-10.0 min. MS Level: 1. Polarity Any. Spectrum type: Profile. Min time span (min): 0.25. Min height: 4E3. *m/z* tolerance: 0.0019 m/z & 10ppm. **Chromatogram deconvolution**: *Algorithm:* Savitzky-Golay. Min peak height: 6E3. Peak duration range (min): 0.32-3.3. Derivate threshold level: 50%. **Isotopic peaks grouper:** *m/z tolerance*: 0.0018 or 10 ppm. *Retention time tolerance*: 0.2 min. *Monotonic shape*: checked. *Maximum charge*: 1. Representative isotope: **Most intense Adduct search**: *RT tolerance:* 0.2 min. Adducts: $[M+Na]^+$, $[M+H]^+$, $[M+H-H2O]^+$, $[M+ACN+H]^+$. *m/z* tolerance: 0.0016 m/z or 9 ppm. Max relative adduct peak height: 20%. **Formula predictor**: *m/z* tolerance: 0.0016 or 6 ppm. Values show in the table correspond to ± 20 % of each element present in the TTX. Phosphorus and sulfur were added in order to test the robustness of the predictor algorithm (Table 1-SI) *Element count heuristics:* all checked. *RDBE restrictions:* checked. Range: -1 to 10. RDBE must be integer. *Isotope pattern filter:* Isotope m/z tolerance: 0.002 or 10 ppm. Minimum absolute intensity: 5.0E1. Minimum score: 85%. *Ms/Ms filter:* Ms/Ms *m/z* tolerance: 0.0016 or 10 ppm. MS/MS score threshold: 80%**. Online database search:** *Database:* PubChem Compound Database. Charge 1. Ionization type: $[M+H]^+$. Number of results: 10. *m/z* tolerance: 0.0019 or 6 ppm. *Isotope pattern filter:* m/z tolerance: 0.0015 or 5 ppm. Minimum absolute intensity 1.0E4. Minimum score: 95.5%

Table S2. Range of element used during Tetrodotoxin dereplication

Element	Minimum number of atoms searched	Maximum number of atoms searched
**Carbon**	9	13
**Phosphorus**	0	1
**Nitrogen**	2	4
**Hydrogen**	13	20
**Oxygen**	7	9
**Sulphur**	0	2

Sulphur and Phosphorus have been included in order to test the robustness of empirical formula predictor.

References

1. Miller, J.N.; Miller, J.C.; Miller, R.D. *Statistics and chemometrics for analytical chemistry*, Seventh edition ed.; Pearson Education Limited: Harlow, United Kingdom, 2018.
